# Activation of TRKA receptor elicits mastocytosis in mice and is involved in the development of resistance to KIT-targeted therapy

**DOI:** 10.18632/oncotarget.18027

**Published:** 2017-05-19

**Authors:** Min Yang, Zengkai Pan, Kezhi Huang, Guntram Büsche, Friedrich Feuerhake, Anuhar Chaturvedi, Danian Nie, Michael Heuser, Felicitas Thol, Nils von Neuhoff, Arnold Ganser, Zhixiong Li

**Affiliations:** ^1^ Department of Hematology, Hemostasis, Oncology, and Stem Cell Transplantation, Hannover Medical School, Hannover, Germany; ^2^ Department of Hematology, Sun Yat-Sen Memorial Hospital, Sun Yat-Sen University, Guangzhou, China; ^3^Institute of Pathology, Hannover Medical School, Hannover, Germany; ^4^ Department of Pediatric Hematology-Oncology, University of Duisburg-Essen, Essen, Germany

**Keywords:** targeted therapy, TRK, KIT, resistance, mastocytosis

## Abstract

The neurotrophins (NTs) play a key role in neuronal survival and maintenance. The TRK (tropomyosin-related kinase) tyrosine kinase receptors (TRKA, TRKB, TRKC) are high affinity receptors for NTs. There is increasing data demonstrating an important role of the TRK family in cancer initiation and progression. NTs have been known for many years to promote chemotaxis, maturation, and survival of mast cells. However, the role of NT signaling in the pathogenesis of mastocytosis is not well understood. In this study, we demonstrate that activation of TRKA by its ligand nerve growth factor (NGF) is potent to trigger a disease in mice with striking similarities to human systemic mastocytosis (SM). Moreover, activation of TRKA by NGF strongly rescues KIT inhibition-induced cell death of mast cell lines and primary mast cells from patients with SM, and this rescue effect can be efficiently blocked by entrectinib (a new pan TRK specific inhibitor). HMC-1 mast cell leukemia cells that are resistant to KIT inhibition induced by TRKA activation show reactivation of MAPK/ERK (extracellular signal-regulated kinase) and strong upregulation of early growth response 3 (EGR3), suggesting an important role of MAPK-EGR3 axis in the development of resistance to KIT inhibition. Targeting both TRK and KIT significantly prolongs survival of mice xenotransplanted with HMC-1 cells compared with targeting KIT alone. Thus, these data strongly suggest that TRKA signaling can improve neoplastic mast cell fitness. This might explain at least in part why treatment with KIT inhibitors alone so far has been disappointing in most published clinical trials for mastocytosis. Our data suggest that targeting both KIT and TRKs might improve efficacy of molecular therapy in SM with KIT mutations.

## INTRODUCTION

Mastocytosis is one subcategory of myeloid neoplasms as defined by the 2016 World Health Organization (WHO) classification of myeloid neoplasms and acute leukemia [1]. Mastocytosis is characterized by a clonal, neoplastic proliferation of morphologically and immunophenotypically abnormal mast cells (MC) accumulating in one or more organ systems [2, 3]. Systemic mastocytosis (SM) is a more aggressive variant associated with extracutaneous involvement that may be accompanied with dysfunction/failure of multiorgan and shortened survival [4, 5]. The KIT D816V mutation can be found in over 80% patients with SM [2]. SM treatment is highly individualized and generally palliative. Allogenic hematopoietic stem cell transplantation may be considered as a viable and potentially curative therapeutic option for advanced SM, but its definitive role remains unclear [5]. Allogenic NK cells can eradicate myeloblasts but not malignant mast cells in SM associated with acute myeloid leukemia [6]. Although KIT inhibitors showed very good inhibitory effects on mast cells *in vitro*, however, treatment with KIT inhibitors alone so far has been disappointing in most published clinical trials for mastocytosis [2, 5, 7–9], probably due to development of resistance to kinase inhibitors [5, 10]. Even in the recently published study demonstrating therapeutic benefit in some patients with advanced SM by targeting KIT, the overall survival is still not satisfying (46% at 3 years) [7]. These findings underscore the need to develop more efficient treatment strategies for patients with SM.

Although KIT mutations, particularly KIT D816V, have been considered as a key mutation for mastocytosis, however, increased body of data indicates that KIT D816V may be a late event in the pathogenesis of mastocytosis and other alterations/mutations may be involved as early events [11]. Identifying such genetic/epigenetic alterations and understanding their interactions and the molecular mechanisms involved in mastocytosis are of the utmost importance for developing rationally targeted therapy. The neurotrophins (NTs: nerve growth factor = NGF, brain-derived neurotrophic factor = BDNF, NT-3, and NT-4) play a key role in neuronal survival and maintenance. The TRK (tropomyosin-related kinase) tyrosine kinase receptors (TRKA, TRKB, TRKC) are high affinity receptors for NTs. The TRK family is emerging as an important player in carcinogenic progression [12, 13]. NTs have been known for many years to promote chemotaxis, maturation, and survival of MCs by preventing apoptotic death [14–16]. For instance, NGF up-regulates expression of human mast cell characteristics (e.g. up-regulation of tryptase) and might have different biological effects at different stages of mast cell development [14]. On the other hand, mast cells synthesize, store, and release NGF. Human mast cell tryptase can cleave pro-NGF, and mature NGF may result from tryptase action [17]. This may fundamentally modify the actions of pro-NGF/NGF [17]. To date, the role of NT signaling in the development of mastocytosis is not well understood. Recently, Peng et al., observed elevated expression of TRK receptors and enhanced neurotrophin levels on mast cells in patients with mastocytosis, and suggested a pivotal role of TRK signaling in the pathogenesis of mastocytosis [15]. Our group has recently reported a potential role of TRK signaling in leukemogenesis [12, 18]. More recently, we reported mastocytosis induced by activation of TRKB in murine hematopoietic stem/progenitor cells *in vivo* [19]. In this study, we demonstrate that activation of TRKA by its ligand NGF is also potent to elicit a disease with striking similarities to human SM in a mouse model and is involved in the development of resistance to KIT-targeted therapy.

## RESULTS

### Mastocytosis induced by activation of TRKA receptor in murine hematopoietic stem/progenitor cells

**Figure 1:**
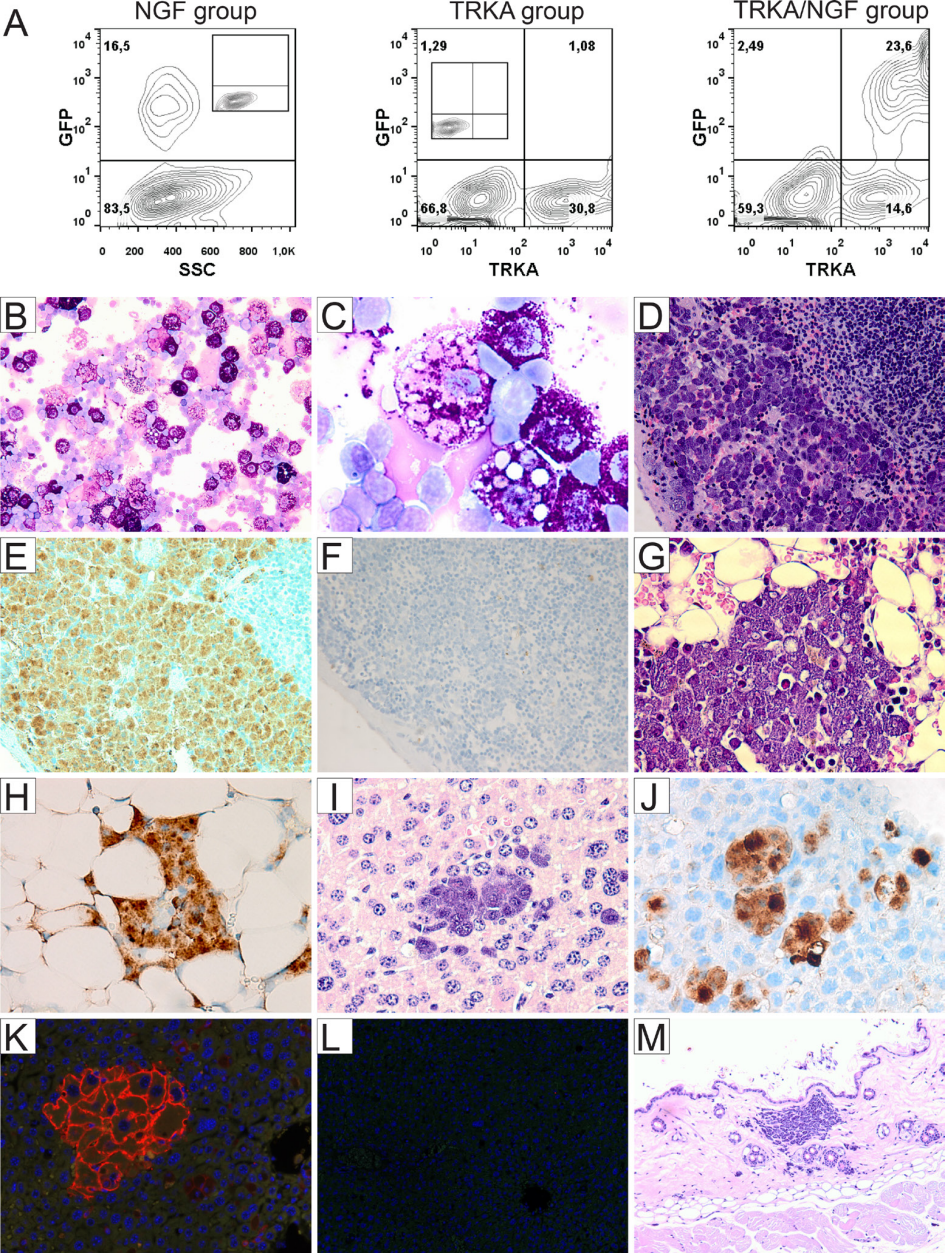
Development of mastocytosis in mice transplanted with TRKA/NGF-modified hematopoietic stem/progenitor cells (**A**) Flow cytometric analysis demonstrating expression of transgenes (NGF and TRKA, measured by enhanced green fluorescent protein and an antibody against TRKA, respectively) after one round of transduction on day of transplantation (negative controls shown as inset). Representative cytology (May-Grünwald-Giemsa), histopathology (H&E), immunohistochemical stains, and immunofluorescence analysis from 3 animals with SM (**B**–**E**, **G** and **H**: mouse #1193; **I**–**K**: mouse #1182; M: mouse #1183). There were multifocal, dense infiltrates of mast cells (≥ 15 mast cells in aggregates) in different organs. (B, C) Cytospins of spleen cells from mouse #1193 showing round (typical), mature mast cells with abundant cytoplasm filled with granules (× 200, × 1000). Note phagocytosis of red cells in some mast cells (C). (D) Accumulation of mast cells in the red pulp of spleen (× 200). Immunohistochemical staining for *CD25* (marker for neoplastic mast cells) showing infiltration of mast cells in spleen (E, brown color, × 200), but no increased mast cells in mouse #1185 with acute leukemia from the TRKA/NGF group (**F**, Supplementay Figure 1). (G) Bone marrow section showing infiltration of mast cells (× 400), highlighted by immunohistochemical stains for *CD25* (H, × 400) and *tryptase* (Supplementary Figure 2). (I) Section of liver showing infiltration of mast cells in mouse #1182 (× 400). Immunohistochemical staining for *CD25* (J, × 400) highlighting infiltration of mast cells in the liver. Negative controls for Figure 1H and 1J shown in Supplementary Figure 1. (K) Immunofluorescence analysis showing TRKA expression on the surface of mast cells, demonstrating that neoplastic mast cells were derived from gene-modified cells (× 400). (**L**) Negative control for (K). (**M**) Section of skin showing infiltration of mast cells in mouse #1183 (× 100).

To investigate the role of TRKA signaling in the pathogenesis of mastocytosis and acute leukemia, 19 C57BL/6J mice were transplanted with retrovially gene-modified primary hematopoietic stem/progenitor cells (TRKA/NGF = 7, TRKA = 6, NGF = 6) in two independent experiments (Figure 1A). In another separate study, seven animals were transplanted with TRKA (*n* = 3) or LNGFR (low-affinity nerve growth factor receptor, *n* = 4) modified cells alone. In the TRKA/NGF group, four animals developed acute leukemia within 6 months after transplantation (Supplementary Figure 1), while three animals developed SM within 12 months after transplantation. Consistent with SM induced by TRKB activation [19], abnormal mast cells mainly showed features of mature hypergranular mast cells (Figure 1B–1D) [20]. These cells expressed *CD25*, *tryptase*, *KIT*, and TRKA (Figure 1, Supplementary Figure 2). SM mainly affected spleen, liver, and bone marrow with multifocal compact mast cell infiltrates (Figure 1). While skin infiltration of mast cells was observed in one animal (Figure 1M), there was no gut infiltration in any of the SM mice (Supplementary Figure 2). Moreover, mouse #1193 also demonstrated elevated level of plasma *tryptase* (67.5 ng/ml). Plasma level of human NGF in mouse #1193 was below 15 pg/ml. This result is broadly in line with previously published data in patients with SM [15]. Of note, there was no evidence of classical mast cell leukemia or other hematological neoplasm in any of SM mice. At the final analysis, SM animals did not show splenomegaly or hepatomegaly, and blood counts were normal in 2 analyzed mice. Moreover, there were no mutations detected in the *KIT* gene in any of SM mice. One out of 9 mice in the TRKA alone group developed a myeloproliferative neoplasm, probably due to mild activation of TRKA by its overexpression and/or endogenous murine NGF, while no other animals with TRKA alone, NGF alone or LNGFR showed SM or other hematological malignancies. In our historic controls of > 100 animals transplanted in similar settings with different genes, e.g. dTRKA, dLNGFR, FLT3 mutants, tCD34, and SV40 LT, no animals developed SM [19, 21]. These data strongly suggest that activation of TRKA by NGF is important for the development of mastocytosis. Although the SM incidence by TRKA activation was lower than by TRKB activation (3/7 = 43% vs. 12/17 = 71% [19]), our data indicate that activation of both TRKA and TRKB by their ligands are more potent than KIT D816V for induction of SM [19], since SM was not induced by retroviral-mediated expression of KIT D816V in similar settings [22]. Furthermore, only 29% of transgenic mice expressing human KIT D816V developed mastocytosis at an old age (> 12 months) [23]. Abnormal mast cells induced by TRKA and TRKB activation mainly demonstrated features of mature mast cells, which is in line with an early report showing induction of a more mature phenotype of immature human mast cells *in vitro* in response to NGF, most probably via activation of the high-affinity NGF receptor expressed on these cells [14].

### Activation of TRKA affected response of malignant mast cells to KIT inhibition

**Figure 2:**
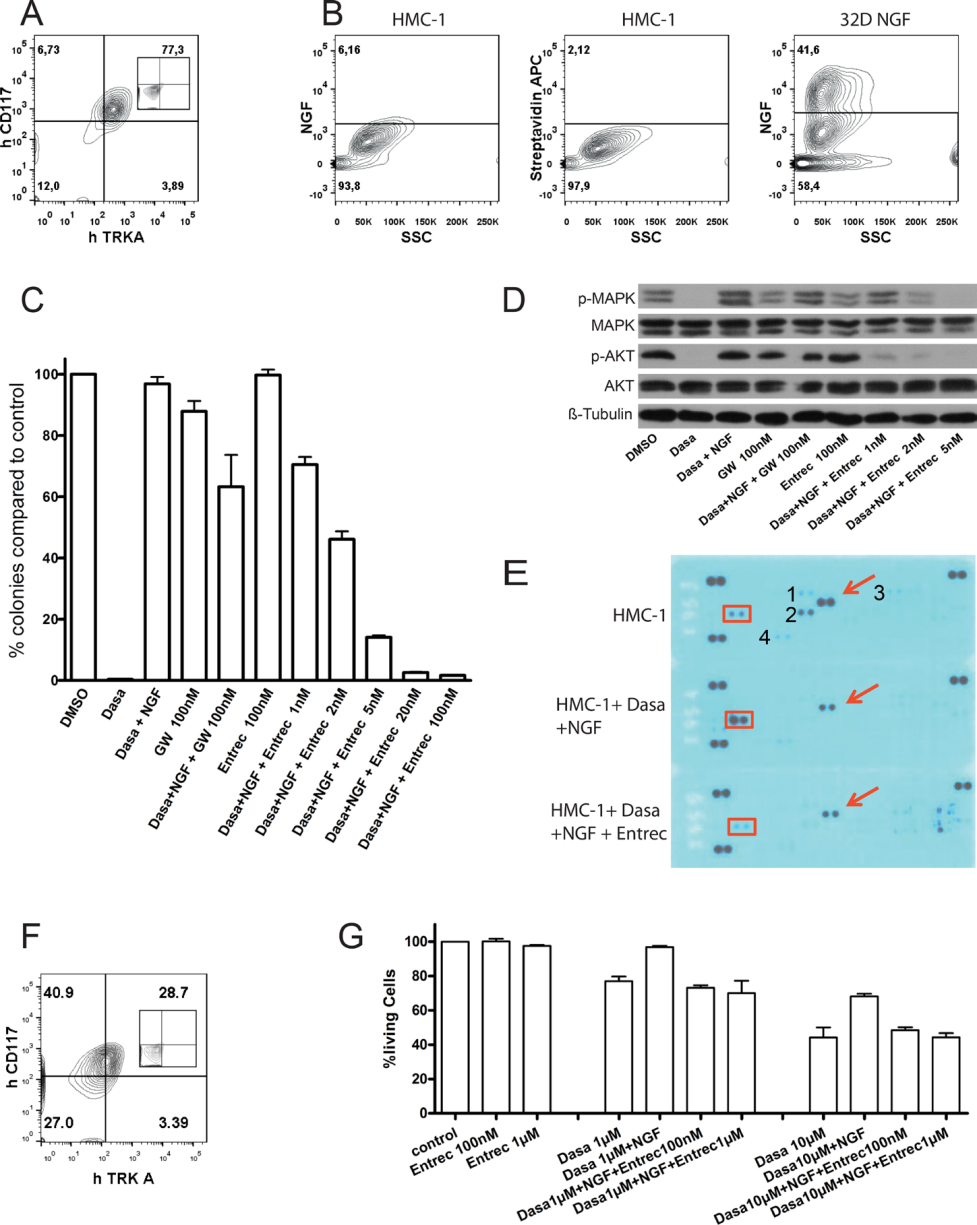
Activation of TRKA affected response of HMC-1 and HMC-1.2 cells to KIT inhibition (**A**) Flow cytometric analysis showing expression of TRKA and KIT in HMC-1 cells carrying only KIT V560G mutation (negative control shown as inset). (**B**) By flow cytometry, HMC-1 cells did not express NGF. Cells were stained with anti-NGF biotin antibody followed by staining with streptavidin APC antibody. 32D cells transduced with retroviral vector expressing NGF were served as a positive control. HMC-1 cells stained with streptavidin APC antibody were used as a negative control. (**C**) To analyze clonal growth of HMC-1 cells, colony forming assay was performed in the presence of kinase inhibitors. Note > 99% growth inhibition by dasatinib = dasa (100 nM, targeting KIT), efficient rescue of HMC-1 cells in the presence of NGF (100 ng/ml), and efficient block (> 98%) of NGF-induced rescue by the new TRK inhibitor entrectinib = entrec (*p <* 0.01 for ≥ 2nM entrec, *p <* 0.00001 for ≥ 5 nM entrec). Dasatinib combination therapy with GW also reduced the number of colonies (*p <* 0.05). Results are presented as the average percentage of colonies formed in the presence of inhibitors (100% value derived from DMSO control). Representative results presented are the mean ± SD (error bars) of at least three independent experiments in duplicates or quadruplicates. (**D**) Immunoblots showing effects of inhibitors and NGF on phosphorylation of AKT and MAPK/ERK. Pro-survival pathways were reactivated by NGF after KIT inhibition and were completely downregulated by 5nM entrectinib. (**E**) A phospho-Receptor PTK array revealed activation of KIT and TRKA in HMC-1 cells. Red boxes and arrows indicated phosphorylation level of TRKA and KIT, respectively. The membranes were exposed and filmed under the same condition. In the array, each receptor PTK is spotted in duplicate. Hybridization signals at the three corners of each array served as positive controls. 1 = FGFR1 (Fibroblast growth factor receptor 1), 2 = VEGFR1 (Vascular endothelial growth factor receptor 1), 3 = Insulin receptor, 4 = RYK. (**F**) Flow cytometric analysis demonstrating expression of TRKA and KIT in HMC-1.2 cells harboring both KIT V560G and D816V mutations (negative control shown as inset). Note much lower expression of TRKA in HMC-1.2 compared with HMC-1 cells. Both HMC-1 and HMC-1.2 cells do not express TRKB or TRKC (data not shown). (**G**) Entrectinib inhibited antiapoptotic effects of NGF-mediated activation of TRKA in HMC-1.2 cells. Results presented are the mean plus or minus SD of at least two independent experiments. NGF concentration was 100 ng/ml. Similar results were observed in colony assays (data not shown).

**Figure 3:**
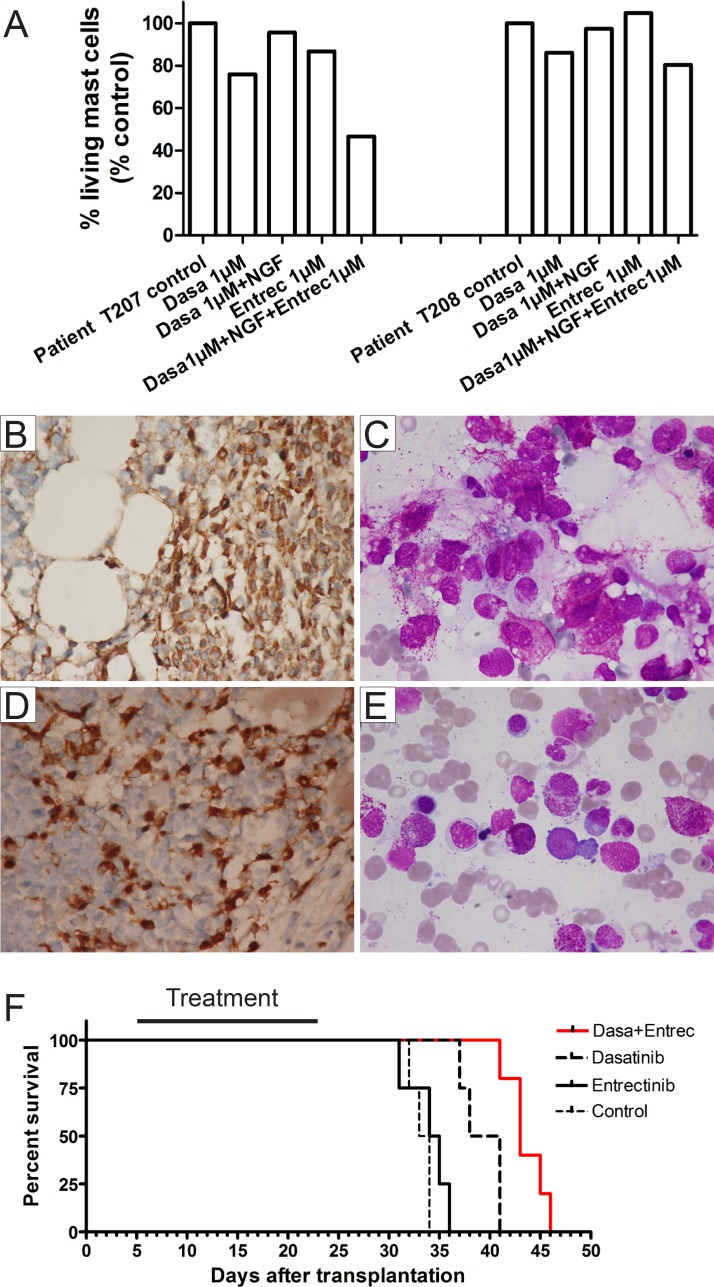
Targeting both TRK and KIT in primary mast cells from patients with SM and in xenotransplanted mice (**A**) Additional inhibition of TRK improved targeted treatment by KIT inhibition in malignant mast cells from patients T207 and T208 *in vitro*. Both patients had KIT D816V mutation and TRKA expression on mast cells. Mast cells were cultured in the presence of inhibitors for 48 hours before apoptosis analysis. Note the strong rescue effect of NGF in patient T207. (**B**–**E**) Immunohistochemical staining for tryptase (B, D, × 600) and bone marrow smears (C, E, × 1000) showing infiltration of mast cells in bone marrow in patients T207 (B, C) and T208 (D, E). T207 = Aggressive SM (the major criterion and at least 3 minor criteria for diagnosis of SM [5] were present), T208 = SM with associated clonal hematological non-mast cell lineage diseases (*SM-AHN,* at least 3 minor criteria for diagnosis of SM were present). (**F**) Kaplan-Meier analysis of animal survival. In line with *in vitro* data, entrectinib alone did not affect the survival of animals compared with the placebo group. Both treatment regimens (dasatinib alone and dasatinib/entrectinib) showed a *significantly prolonged survival* of NSG animals transplanted with HMC-1 cells (*p* < 0.0002 for both regimens vs. placebo and entrectinib), while treatment with entrectinib in combination with dasatinib was associated with a prolongation of survival times by a further 4.4 ± 1.2 days (*p* < 0.003). There were 17 animals transplanted: control/placebo = 4, entrectinib = 4, dasatinib = 4, dastinib/entrectinib = 5. Black bar denotes treatment period.

Since NTs are important for survival of MCs and treatment with KIT inhibitor (in particular imatinib and dasatinib) alone did not lead to a durable response in most patients with mastocytosis, we investigated whether TRKA activation improves neoplastic mast cell fitness in the presence of mutations of KIT and is involved in resistance of mast cells with KIT mutations to KIT inhibitors. To this end, we took advantage of the human mast cell leukemia cell line HMC-1 with KIT V560G mutation and TRKA expression, but no detectable NGF by flow cytometric analysis (Figure 2A, 2B). An earlier study demonstrated expression of NGF mRNA in HMC-1 cells. However, in that study constitutive activation of TRKA and c-FOS was only observed when cells were cultured with NGF, suggesting that the level of *NGF secreted,* if any, was very low [24]. In colony assays and liquid culture, TRKA activation by NGF efficiently rescued HMC-1 cells from cell death mediated by KIT inhibition. This rescue effect by NGF was efficiently inhibited by entrectinib (a new TRK specific inhibitor [13]) (Figure 2C, Supplementary Figure 3). Of note, entrectinib at the concentration of ≥ 2 nM had more potent capability to block NGF-induced rescue than the old TRK inhibitor GW-441756. Entrectinib efficiently inhibited colony formation of HMC-1 cells at nanomolar concentration (∼100 nM) that is well below the concentration safely achieved in humans [25]. Interestingly, MAPK/ERK (extracellular signal-regulated kinase) and AKT were reactivated by NGF after KIT inhibition and were completely downregulated by 5nM entrectinib (Figure 2D). Of note, activity of AKT and ERK pathways was correlated with phosphorylation levels of KIT and TRKA (Figure 2E). After treatment with dasatinib (100 nM, 90 min) and stimulation with NGF (100 ng/ml, 30 min), the phosphorylation of KIT was strongly decreased, while the phosphorylation of TRKA was stimulated > 3-fold. Entrectnib treatment (100 nM, 90 min) strongly induced dephosphorylation of TRKA. We did not detect NGF in the HMC-1 cells by flow cytometry, but TRKA was still activated, probably due to TRKA overexpression and/or very low *NGF secreted by the cells*. VERFR1, FGFR1, RYK, and insulin receptor were also activated in HMC-1 cells, but almost completely dephophorylated after dasatinib treatment (Figure 2E), indicating that these receptors are unlikely mediators for resistance to KIT inhibition. Importantly, TRKA activation also affected the response of both HMC-1.2 cells harboring KIT D816V mutation and primary mast cells isolated from patients with SM associated with KIT D816V to KIT-targeted therapy (Figures 2F, 2G, 3A–3E, Supplementary Figure 4). The rescue effect of NGF in HMC-1.2 cells and cells from patient T208 was not as strong as in the HMC-1 cells and patient T207, probably due to low expression of TRKA in these cells (around 30%, Figure 2F). Patients T207 and T208 showed heterogenic response to combined therapy, indicating different response sensitivities among patients. In line with previously published data [15], plasma levels of NGF in the two patients (T207 and T208) were below 15 pg/ml. Of note, SF3B1 and SRSF2 were mutated in patient T208, while there was no mutation found in any of 22 tested genes associated with myeloid neoplasms including ASXL1, BRAF, DNMT3A, FLT3, NRAS, KRAS, SF3B1, and SRSF2 in patient T207. This suggests that TRKA activation may be an important factor for improved neoplastic mast cell fitness in the patient T207.

It has been shown that KIT and TRK activation not only induce common signaling pathways, but also induce unique signal cascades [26]. Dutta et al. recently reported that NGF could support long-term survival of HMC-1 cells in the presence of imatinib [26]. They identified 117 genes upregulated by NGF stimulation in HMC-1 cells, among these genes 58 genes were also downregulated by KIT inhibition, while 59 genes were specifically affected only by NGF stimulation [26]. To examine differences between TRK and KIT signaling pathways and to understand molecular pathways responsible for resistance to KIT inhibition, we performed qRT-PCR in the treated HMC-1 cells. We analyzed expression of KLF2 (kruppel-like factor 2), EGR1 (early growth response 1) (genes upregulated by both KIT and TRK reported by Dutta et al. [26]), EGR3 (early growth response 3) [27] and GDF15 [28] (affected specifically by NGF stimulation [26]). Interestingly, EGR3 was 21.1-fold downregulated in HMC-1 cells by dasatinib treatment. Dasatinib treatment followed by NGF stimulation induced a 1269.4-fold increased expression of EGR3 compared with dasatinib treated cells. Additional treatment with entrectinib brought EGR3 expression down to the expression level in cells treated by dasatinib alone (Supplementary Table 1). We also observed slight differences of EGR1 expression by dasatinib and by NGF stimulation, while there was no difference regarding expression of GDF15 and KLF2 induced by TRK and KIT activation (Supplementary Table 1). Of note, EGR transcriptional regulators (e.g. EGR3) are examples of MAPK-ERK regulated genes [27]. Taken together, our data suggest that TRKA activation mediates resistance to KIT inhibition likely via the MAPK-EGR3 axis.

Finally, we wished to test the combined therapy with dasatinib and entrectinib *in vivo* to evaluate how effective this combination may be in a more real-life situation. Dasatinib demonstrated an IC50 value of approximately 5 µM for HMC-1.2 cells in *in vitro* cytotoxicity assays (Figure 2G and data not shown), therefore, xenotransplantation of HMC-1.2 cells into NOD-*scid IL2rγ*^null^ (NSG) mice was not a good model for *in vivo* treatment with dasatinib (max. plasma concentration in mice: around 2µM [29]). Thus, we transplanted HMC-1 cells in to NSG mice and treated the mice with different regimens: placebo, dasatinib alone, entrectinib alone and dasatinib/entrectinib. Importantly, combined therapy with dasatinib and entrectinib significantly prolonged the survival of NSG mice transplanted with HMC-1 cells compared with other groups (Figure 3F), confirming the *in vitro* data (Figure 2C). Of note, murine beta-NGF shares approximately 90% homology at the amino acid level with human beta–NGF and can activate human TRKA [30]. Importantly, combination therapy with dasatinib and entrectinib was well tolerated and did not promote weight loss. Although the KITV560G mutation seen in HMC-1 cells is not common in patients with SM, our data provide the first proof-of-concept for targeting both KIT and TRK pathways in SM. Collectively, our data strongly suggest that TRKA signaling can improve neoplastic mast cell fitness. This might explain at least in part why treatment with KIT inhibitors alone so far has been disappointing in most patients with mastocytosis [2]. Since NGF is also expressed by bone marrow stroma cells [12, 31], it might activate TRKA and protect mast cells from cell death induced by KIT inhibition. Inhibition of TRKA activation may overcome resistance to KIT-targeted therapy in SM patients. Moreover, activation of TRKB in HMC-1 cells with ectopic expression of TRKB also very efficiently rescued HMC-1 cells from KIT inhibition, and this effect was successfully blocked by entrectinib (Supplementary Figure 5). Of note, TRKB and TRKC are also expressed by neoplastic mast cells [15], and mast cells express mRNA for all NTs and release at least active NGF, NT-3 (can activate all TRKs), and NT-4 (both BDNF and NT-4 can activate TRKB) [16]. Thus, our data suggest that other members of the TRK family may be also involved in the development of resistance to KIT inhibition.

## DISCUSSION

Here, we for the first time demonstrate SM development by activation of TRKA *in vivo* , although the number of analyzed mice was limited, and present a new example that cancer cells can develop resistance to the therapeutic inhibition of receptor tyrosine kinase signaling by ligand-mediated receptor activation [32], which provide a means for cancer cells to re-establish the signaling pathways (e.g. MAPK in this study) that were present before the inhibitor therapy. Since NTs can be released by mast cells as well as bone marrow stromal cells, our data, together with our recent report demonstrating SM induction by BDNF-mediated activation of TRKB [19], suggest an important role of autocrine and/or paracrine activation of TRKs in the pathogenesis of mastocytosis. Moreover, we show a significantly prolonged survival of mice xenotransplanted with HMC-1 cells by targeting both TRK and KIT compared with targeting KIT alone. Our data might have significant implications for clinical practice. Efficient targeting of both KIT and TRKs might improve the efficacy of molecular therapy in SM patients with KIT mutations. Of note, the new pan-TRK inhibitors entrectinib and LOXO-101 are now being tested in clinical trials in patients with solid tumors and have already yielded dramatic clinical activity in some patients, demonstrating that some human cancers are TRK dependent [13, 25, 33, 34], and TRKs may be good targets for molecular therapy.

The majority of adult patients with systemic mastocytosis (SM) has aberrant mast cell morphology such as spindle shapes and hypogranulation, and harbor the KIT D816V mutation [3, 4, 35]. However, a subgroup of patients (< 10% of all SM) show mature mast cells morphology (round, fully granulated) [20, 35–37]. Patients with such so-called well-differentiated systemic mastocytosis (WDSM) harbored low frequency of KIT D816V mutation compared with other forms of SM (29% vs. 93%) [35]. A recently proposed classification suggests a diagnosis of chronic mast cell leukemia (MCL, defined by at least 20% MCs on bone marrow smears and absence of C-Findings) for patients with MCL in whom the condition is less aggressive and does not induce organ damage within a short time [38]. Interestingly, mast cells in patients with chronic MCL exhibit a more mature morphology and likely carry lower KIT mutations when compared to acute MCL [38]. In our mouse models, the mast cell disease induced by TRKA/NGF and TRKB/BDNF [19] is strikingly similar to human SM, particularly to WDSM. The criteria for chronic MCL can be fulfilled in at least three TRKB/BDNF and TRKA/NGF mice (at least 20% MCs on spleen smears and absence of C-Findings). Of note, spleen were affected in all animals with SM in our models, while bone marrow and liver were only variably involved, and some animals even did not have infiltration of mast cells in bone marrow. Interestingly, mast cells induced by TRKA/NGF and TRKB/BDNF strongly expressed *CD25* (Figure 1) [19], while *CD25* expression was generally not detectable in WDSM patients. Importantly, we did not detect any mutation in the *KIT* gene in any of analyzed mice with SM, indicating that contribution of mutated *KIT* signaling to SM development in our model is unlikely. Although not all aspects of the human condition are reproduced in our models, our data indicate that TRKA and TRKB activation may play an important role in pathogenesis of SM, particularly in development of WDSM and/or chronic MCL. Targeting TRKs in such patients might be an attractive therapy. It will be interesting to investigate whether activation of TRKC can also induce SM. KIT mutation alone does not explain the full clinical spectrum of SM, and new data suggested that KIT D816V is a late event in the pathogenesis of SM [11]. Moreover, animal studies suggest that cooperating events are *required* for KIT D816V mutation in induction of SM [22, 23, 39]. It will be important to determine whether TRK signaling cooperates with KIT mutation in induction of SM.

Why was the SM incidence after TRKA activation lower than after TRKB activation (43% vs. 71% [19])? The reason for this difference is unclear. However, some data indicate that different NTs can induce different signaling upon activating the same TRK receptor in neuronal cells. BDNF, NT-3 and NT-4 can activate TRKB, while TRKA can be activated only by NGF and NT-3. It is obvious that BDNF and NT-4 have distinct as well as overlapping actions [40, 41]. The distinct activities of NT-4 and BDNF may result partly from differential activation of TRKB and its down-stream signals [41]. Moreover, it appears that TRKA and TRKB are fundamentally different in their signaling capabilities in neurons [42]. TRKA may use a single signaling pathway to induce responses such as survival, while TRKB may utilize multiple signal pathways. Whether such a difference between TRKA and TRKB is also present in hematopoietic cells remains to be determined. Interestingly, all TRK receptors (TRKA, TRKB, TRKC) are expressed on LAD2 cells (Yang and Li, unpublished data), another human mast cell line [43]. It is important to examine the impact of different NTs and coexpression of ≥ 2 TRK receptors on development of SM, survival and growth of malignant mast cells.

The molecular mechanisms by which TRK signaling contributes to SM remain to be determined. ERK signaling seems to be important for functions of mast cells and might be involved in mastocytosis development [44, 45]. A deleted form of TRKA (ΔTrkA), in which 75 amino acids are lacking in the extracellular domain, was identified in an AML patient [46]. We demonstrated a strong leukemogenic activity of ΔTrkA [47]. Of note, none of > 20 C57Bl/6J transplanted with ΔTrkA modified primary Lin-cells developed SM [47, 48], while SM was observed in 43% of mice transplanted with TRKA/NGF in the present study. Interestingly, ΔTrkA did not activate MAPK/ERK [47], while a strong activation of ERK was observed by TRKA activated by NGF [47] and in HMC-1 cells (Figure 2D). Moreover, efficient growth inhibition of HMC-1 cells was associated with downregulation of MAPK/ERK (Figure 2D). These data strongly suggest that ERK might be important for mastocytosis development and survival of malignant mast cells. MAPK-ERK signaling is also upstream of EGR transcriptional regulators (e.g. EGR3) [27]. In the present study, we observed very high upregulation (> 1200-fold) of EGR3 after dasatinib treatment and NGF stimulation in HMC-1 cells, that were resistant to KIT inhibition. Our data suggest that EGR3 may represent a major difference between TRK and KIT signaling, and the MAPK-EGR3 axis may play an important role in development of resistance to KIT inhibition. Dutta et al. observed only a 22.8-fold upregulation of EGR3 in HMC-1 cells after NGF stimulation [26]. This difference may be due to experiment set up (serum starvation for 4h in the present study vs. 17h in their study) and analysis methods used (quantitative RT-PCR vs. microarray analysis). Of note, EGR3 plays an important role in neuronal functions [49, 50], muscle stretch receptor function, angiogenesis [51], and immunity (e.g. controlling proliferation of thymocytes) [52]. EGR3 is highly expressed in some cancer, such as prostate tumors [53], but its role in tumorigenesis and drug resistance remains largely unknown. Thus, further studies are needed to investigate the role of MAPK/ERK and EGR3 in the pathogenesis of mastocytosis and the development of resistance to KIT inhibition in more details. Interestingly, activation of TRKA and TRKB in hematopoietic stem/progenitor cells induced 2 different phenotypes *in vivo*, i.e. SM and acute leukemia. This might be due to different cell populations targeted [54], although other factors might also be involved. Interestingly, SM was induced only in mice transplanted with high expression of TRKA/NGF, suggesting that gene dose might have impact on different phenotypes induced by TRK. Consistent with TRKB activation [19], transplantation of TRKA and NGF modified 32D cells (murine myeloid progenitors) induced only myeloid leukemia in C3H/HeJ animals without any sign of increased mastocyte numbers [12]. Mastocytosis was triggered only when TRKA or TRKB was activated in hematopoietic stem/progenitor cells, strongly supporting the view that mast cells are derived from hematopoietic stem cells [55].

Together with our recent report [19], our data strongly support the findings by Peng et al. [15], who demonstrated enhanced neurotrophin levels and elevated expression of TRK receptors on skin and gut mast cells in patients with mastocytosis, and suggest that TRK signaling may contribute to the pathogenesis of mastocytosis and development of resistance to targeted therapies in SM. Our data suggest an important role of MAPK-EGR3 axis in the development of resistance to KIT inhibition. Targeting TRK signaling might overcome resistance to KIT-targeted therapy in SM. Although we used a retroviral vector expressing NGF in this study, which generally allows high expression of transgenes in hematopoietic stem/progenitor cells, plasma level of human NGF in mouse #1193 with SM was below 15 pg/ml. This is broadly in line with the level in the SM patients from our group and others [15]. This suggest that our mouse model might be suitable for resembling certain types of human mastocytosis *in vivo* and might be useful for further studies to understand molecular mechanisms for development of SM and to test new therapeutic approaches in preclinical settings.

## MATERIALS AND METHODS

### Retroviral transductions and *in vivo* tumorigenesis assays

Retroviral vectors had identical control elements (SFFVp LTR, MESV leader) [12, 56].We isolated hematopoietic stem/progenitor cells enriched lineage negative (Lin^-^) bone marrow cells from C57BL/6J.Ly5.2 mice and performed retroviral transduction as previously described [56]. Co-expression of TRKA/NGF was achieved by co-transduction of 2 different retroviral vectors (TRKA or NGF-IRES-EGFP) into identical target cells (Figure 1A) [56]. Gene-modified cells were then transplanted by tail vein injection into lethally irradiated syngeneic recipients (aged 8–16 weeks). Around 30% of transduced cells were still Lin- (normally < 2% in non-enriched whole bone marrow cells) on day of transplantation, indicating good transduction protocol reserving activity of stem/progenitor cells, which allowed good engraftment of transplanted mice and long-term observation of this study (termination on 424 days after transplantation). Mice were monitored at least in 4 days/week, if needed daily, for tumor-related signs. All animals were obtained from the animal laboratories of Hannover Medical School (MHH) and/or Charles River (Sulzfeld, Germany) and were kept in the animal laboratories of MHH. Animal experiments were approved by the local ethical committee and performed according to their guidelines. SFFVp = polycythemic strain of the spleen focus-forming virus; LTR = long terminal repeat; MESV = murine embryonic stem cell virus; IRES = internal ribosomal entry site; NGF = nerve growth factor; EGFP=enhanced green fluorescent protein.

### Tumor phenotyping

Moribund mice were killed for necropsy, or analyzed when found dead before onset of autolysis [19]. Mice were macroscopically examined for pathological abnormalities during dissection. Enlarged organs were weighed. BM, spleen, liver, skin, gut, kidney, lung, brain and thymus were fixed in a buffered 4% formalin solution and embedded in Paraplast plus (Kendall, Mansfield, MA, USA). Sections were routinely stained with hematoxylin and eosin. Immunohistochemical stains for *tryptase* and *CD25* were performed using BenchMark Ultra (TM) staining machine (Roche, Mannheim, Germany) in selected animals. Blood cell counts were *measured* by using an automatic analyzer (ABC Counter, Scil, Viernheim, Germany).

### Immunofluorescence analysis

Immunofluorescence analysis for detection of TRKA was performed using a purified polyclonal goat antibody against recombinant human TRKA (R&D Systems, Minneapolis, MN) after heat antigen retrieval in citrate buffer at pH6, followed by Alexa 594-conjugated anti-goat secondary antibody (Thermo-Fisher, Waltham, MA). Visualization was performed using the Mantra^®^ image acquisition and Inform^®^ image analysis system (Perkin-Elmer, Waltham, MA).

### Isolation of primary mast cells and DNA sequencing

Primary mast cells were isolated from bone marrow of patients T207 and T208 with SM over Ficoll. In patient T207, the following 23 genes were analyzed for mutations: ASXL1 (exon 12), BRAF (exon 15), CALR (exon 9), CBL (exon 8, 9), CSF3R (exon 14, 17), DNMT3A (exon 11-23), EZH2 (exon 5–8, 14–20), FLT3 (exon 14, 15, 20), IDH1 (exon 4), IDH2 (exon 4), JAK2 (exon 12, 14), KIT (exon 8, 10, 11, 17), KRAS (exon 2, 3), MPL (exon 10), NPM1 (exon 12), NRAS (exon 2, 3), RUNX1 (exon 3-8), SETBP1 (exon 4), SF3B1 (exon 14, 15), SRSF2 (exon 1), TET2 (exon 3–11), TP53 (exon 2–11), and U2AF1 (exon 2, 6). In patient T208, we analyzed 10 genes including ASXL1, DNMT3A (exon 23), IDH1, IDH2, KIT (exon 8, 11, 17), RUNX1, SF3B1, SRSF2, TP53 (exon 5–8), and U2AF1.

### Cell growth in methylcellulose and apoptosis assay

To analyze clonal growth, HMC-1 cells and HMC-1.2 (purchased from Millipore) were plated in H4230 media (StemCell Technologies, Vancouver, Canada) in the presence of kinase inhibitors. The cells were plated as duplicates or quadruplicates. HMC-1 cells with ectopic expression of TRKB were generated by retroviral transduction using a vector expressing human TRKB [12]. We performed PCR for detection of mycoplasma in cell cultures [57] and did not detect contamination of mycoplasma in tested cell lines. Primary mast cells were cultured in the presence of inhibitors for 48 hours before apoptosis analysis. Cell viability was analyzed using the Annexin-V assay (BD Pharmingen, Heidelberg, Germany). Inhibitors dasatinib and entrectinib were purchased from Selleckchem (Houston, TX). NGF and BDNF were purchased from PeproTech. We chose dasatinib for KIT inhibition in this study, since dasatinib treatment showed benefit in some patients with SM [5, 58], and dasatinib is currently being tested in our Phase III clinical trial for CBF AML including KIT D816V mutated patients (ClinicalTrials.gov number, NCT02013648).

### Western blot analysis and antibody array

For signal transduction analysis, cell extracts were prepared following established protocols [47]. Cell lysates were used as indicated in the Figure 2. Antibodies were purchased from Cell signaling Technology (Danvers, Massachusetts). Human Phospho-Receptor PTK arrays were performed following the manufacturer’s instruction (R&D, Minneapolis, MN).

### Enzyme-linked immunosorbent assays (*ELISA*)

Measurement of *tryptase* and human NGF were performed following the manufacturer’s instruction (eBioscience, mouse *MCPT*-*1* Ready-SET-Go!^®^ kit; R&D, human NGF DuoSet kit).

### Growth factor stimulation, RNA-isolation, and qRT-PCR

HMC-1 cells were serum starved for 4 h, then treated with 100nM dasatinib or dasatinib/100 nM entrectinib for 4 hours prior to stimulation with 100 ng/ml NGF. After 120 min stimulation, RNA was isolated by using RNeasy Minin kit (QIAGEN, Hilden, Germany). Reverse transcription was performed using reverse transcriptase kit following the manufacturer’s instruction. Quantitative (TaqMan) RT-PCR was carried out as previously described [59]. TaqMan probes were purchased from Applied Biosystems.

### Xenograft studies

For the xenograft studies, NSG mice were irradiated with 2.5 Gy and transplanted with HMC-1 cells. We chose 10^6^ HMC-1 cells for transplantation, since animals transplanted with ≥ 10^6^ cells became moribund or died around 30 days after transplantation in our preliminary study. Entrectinib was dosed BID, 15 mg/kg, 4 days on, 3 days off per week (http://ignyta.com). Entrectinib was reconstituted and prepared as previously published [60]. In line with published data [60], treatment with entrectinib in nontransplanted NSG mice in our laboratory led to max. plasma concentration of 5100 nM. Dasatinib was dosed BID 10 mg/kg as reported [61]. Dasatinib and entrectinib were given by a gavage. Treatment was started at day 5 after transplantation for 19 days.

### Ethics approval and consent to participate

This study was approved by the ethical committee at the Hannover Medical School.

### Statistical analysis

Regression analysis was performed to compare the impact of different treatment regimens on the likelihood of survival of animals. The results were controlled by Kruskal-Wallis test. A single multivariate stepwise regression analysis was used to compare inhibition efficiency of the colony formation in Figure 2C. *P* values less than 0.05 were considered statistically significant.

## SUPPLEMENTARY MATERIALS FIGURES AND TABLE


