# Supplementary material for: Activation of TRKA receptor elicits mastocytosis in mice and is involved in the development of resistance to KIT-targeted therapy
Source: Oncotarget. 2017 May 19;8(43):73871–83. doi: 10.18632/oncotarget.18027 (PMC5650308; doi:10.18632/oncotarget.18027)
Supplement: Supplementary file 1 [file oncotarget-08-73871-s001.pdf]

Activation of TRKA receptor elicits mastocytosis in mice and is involved in the development of resistance to KIT-targeted therapy

SUPPLEMENTARY MATERIALS

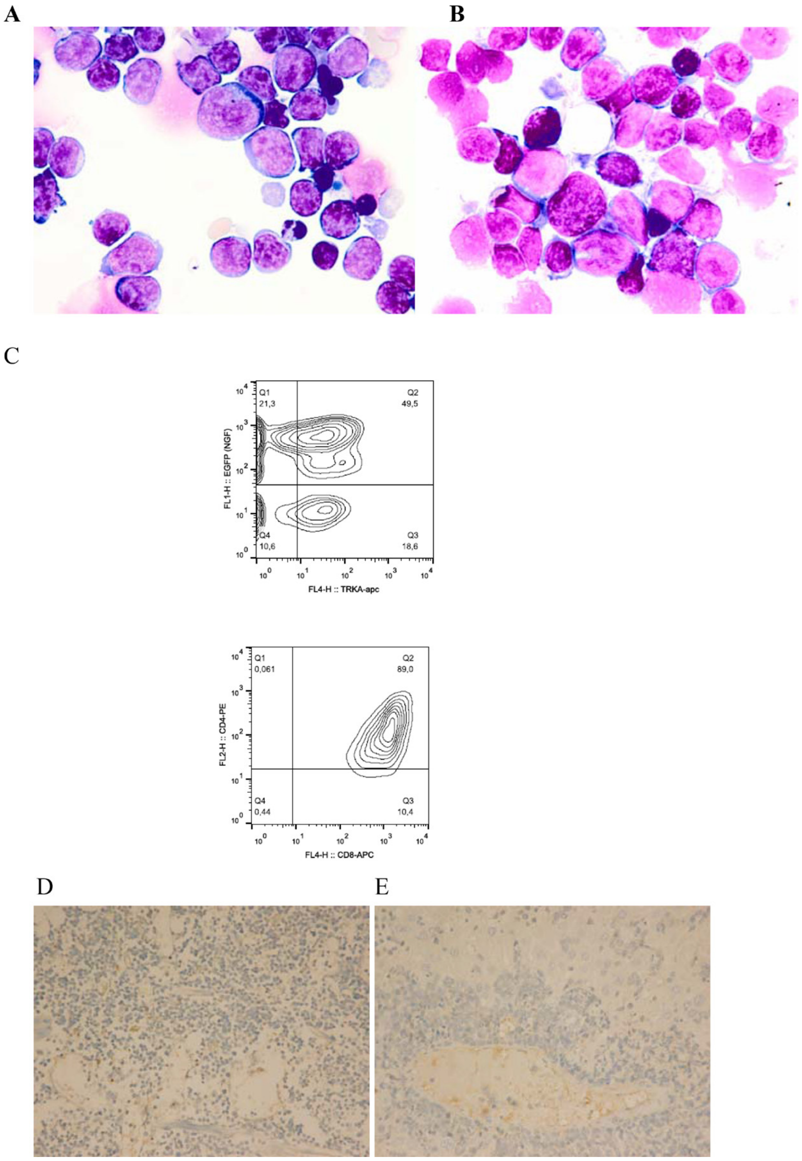

Supplementary Figure 1: Leukemia development by activation of TRKA. In the TRKA/NGF group, four animals developed acute leukemia (1 acute myeloid leukemia, 1 acute lymphoblastic leukemia = ALL, 1 biphenotypic leukemia, 1 unclassifiable) within 6 months after transplantation. Bone marrow cytospin (**A**) and liver cytospin (**B**) from mouse #1185 with ALL showing lymphoblasts (× 1000). (**C**) The leukemic cells were positive for TRKA, NGF, CD4, and CD8, but negative for CD19, CD11b, Gr1, and Ter119 (data not shown). (**D**, **E**) Immunohistochemical staining for *CD25* (marker for neoplastic mast cells) showing no increased mast cells in bone mouse (D) or liver (E) (× 400).

A

B

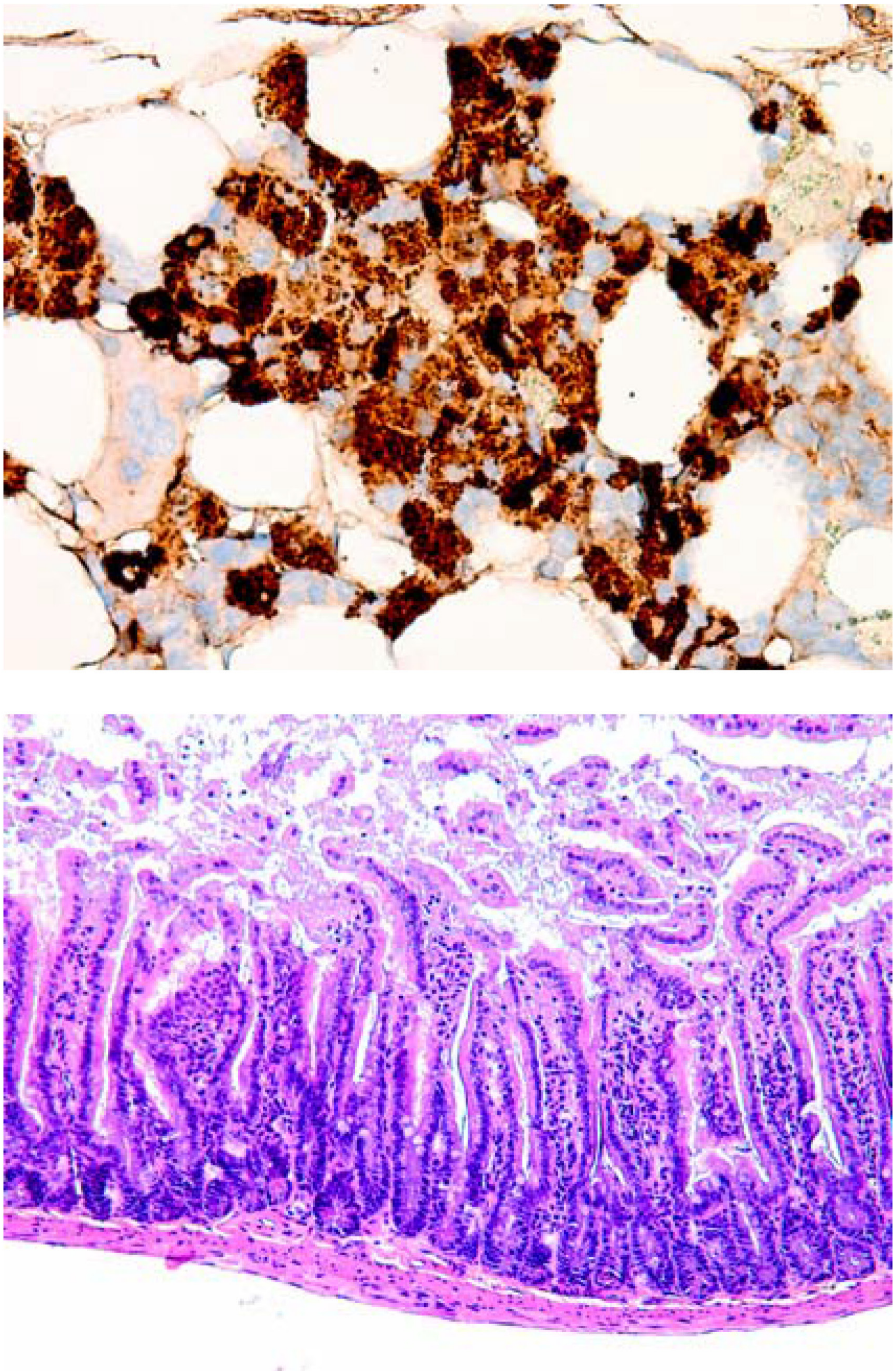

Supplementary Figure 2: Development of mastocytosis in mouse #1193 transplanted with TRKA/NGF-modified hematopoietic stem/progenitor cells. (**A**) Immunohistochemical staining for *tryptase* (brown color, × 400) highlighting infiltration of mast cells in the bone marrow. Elevated level of plasma *tryptase* was observed in mouse #1193 (67.5 ng/ml), while no plasma was available in other 2 animals with SM. Level of plasma *tryptase* was under the detection threshold in control mice #1186 (TRKA/NGF group, T-ALL) and #1207 (NGF group). (**B**) No infiltration of mast cell in the gut.

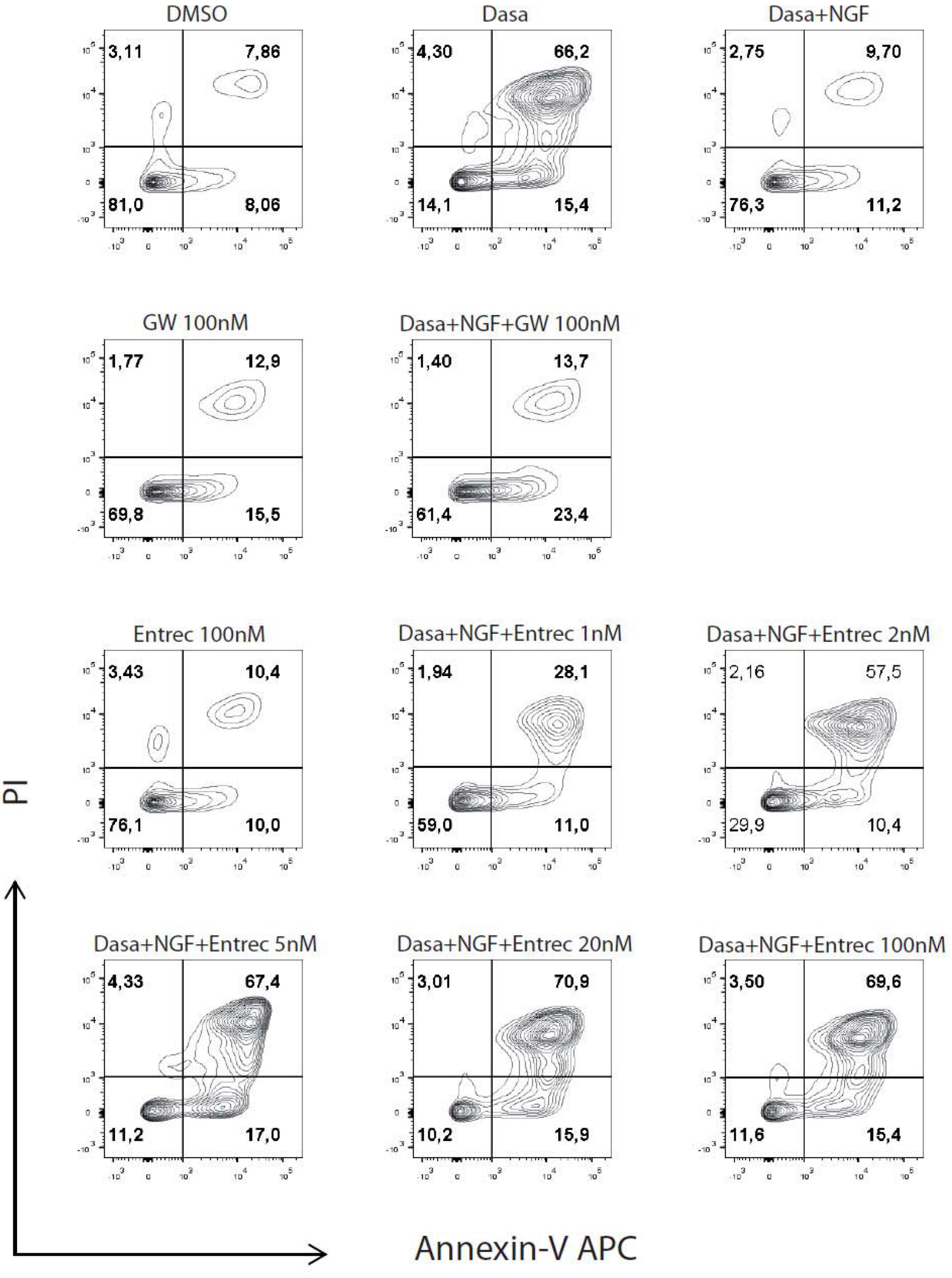

Supplementary Figure 3: Flow cytometry diagrams showing apoptosis of HMC-1 cells after 48 h treatment of inhibitors and NGF stimulation. Cell viability was analyzed using the Annexin-V assay. Annexin V^{+}/PI^{-} = cells in early stage of apoptosis, Annexin V^{+}/PI^{+} = cells in late-stage of apoptosis. This result was consistent with the data obtained with colony assays (Figure 1). Note entrectinib efficiently induced apoptosis after dasatinib treatment and NGF (100 ng/ml) stimulation. Similar data was observed in HMC-1 cells when imatinib was used to inhibit KIT (data not shown). Dasa = dasatinib, 100 nM, Entrec = entrectinib, DMSO = dimethyl sulfoxide.

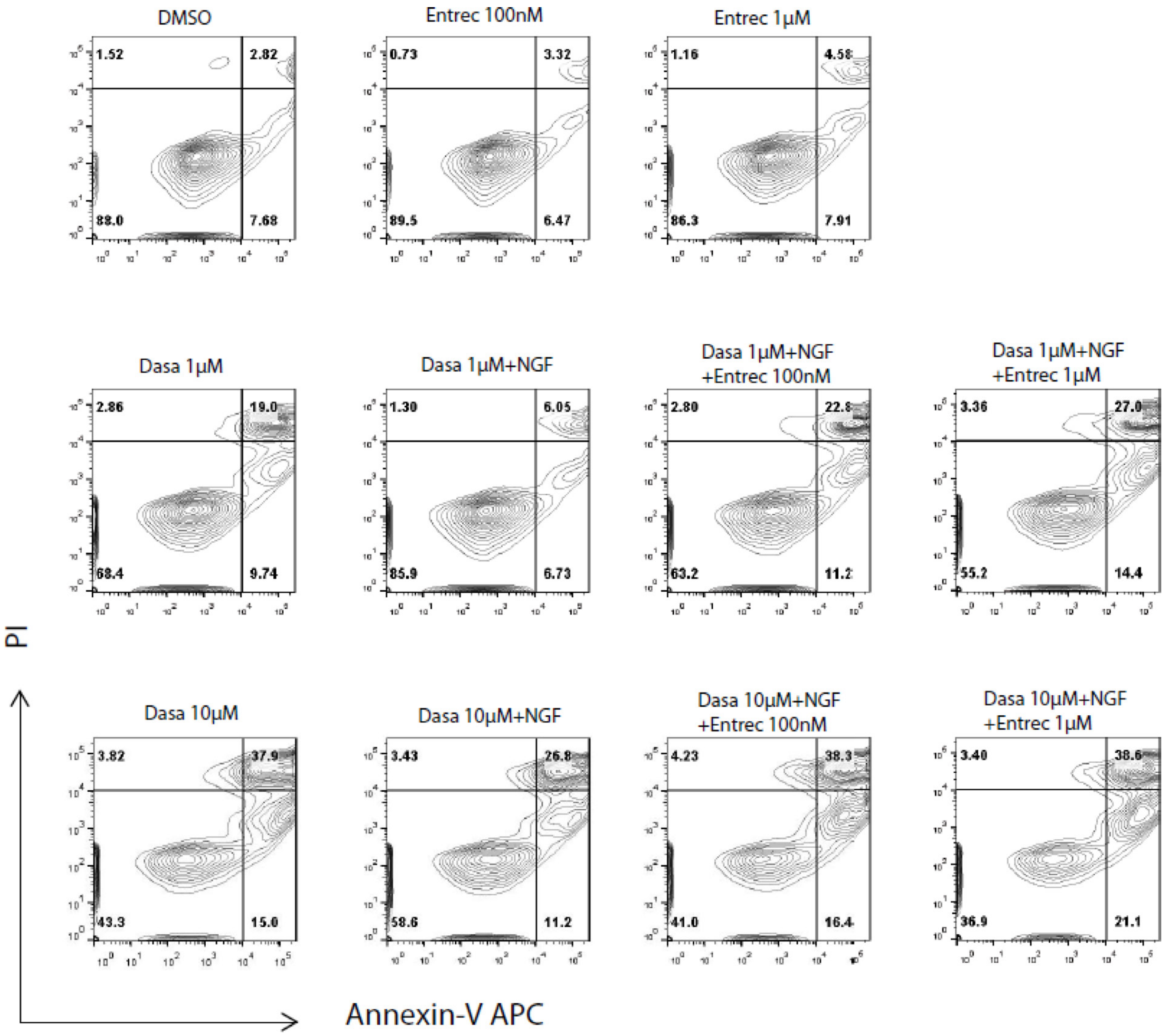

Supplementary Figure 4: Flow cytometry diagrams showing apoptosis of HMC-1.2 cells after 48 h treatment of inhibitors and NGF (100 ng/ml) stimulation. Cell viability was analyzed using the Annexin-V assay. Dasa = dasatinib, Entrec = entrectinib, DMSO = dimethyl sulfoxide.

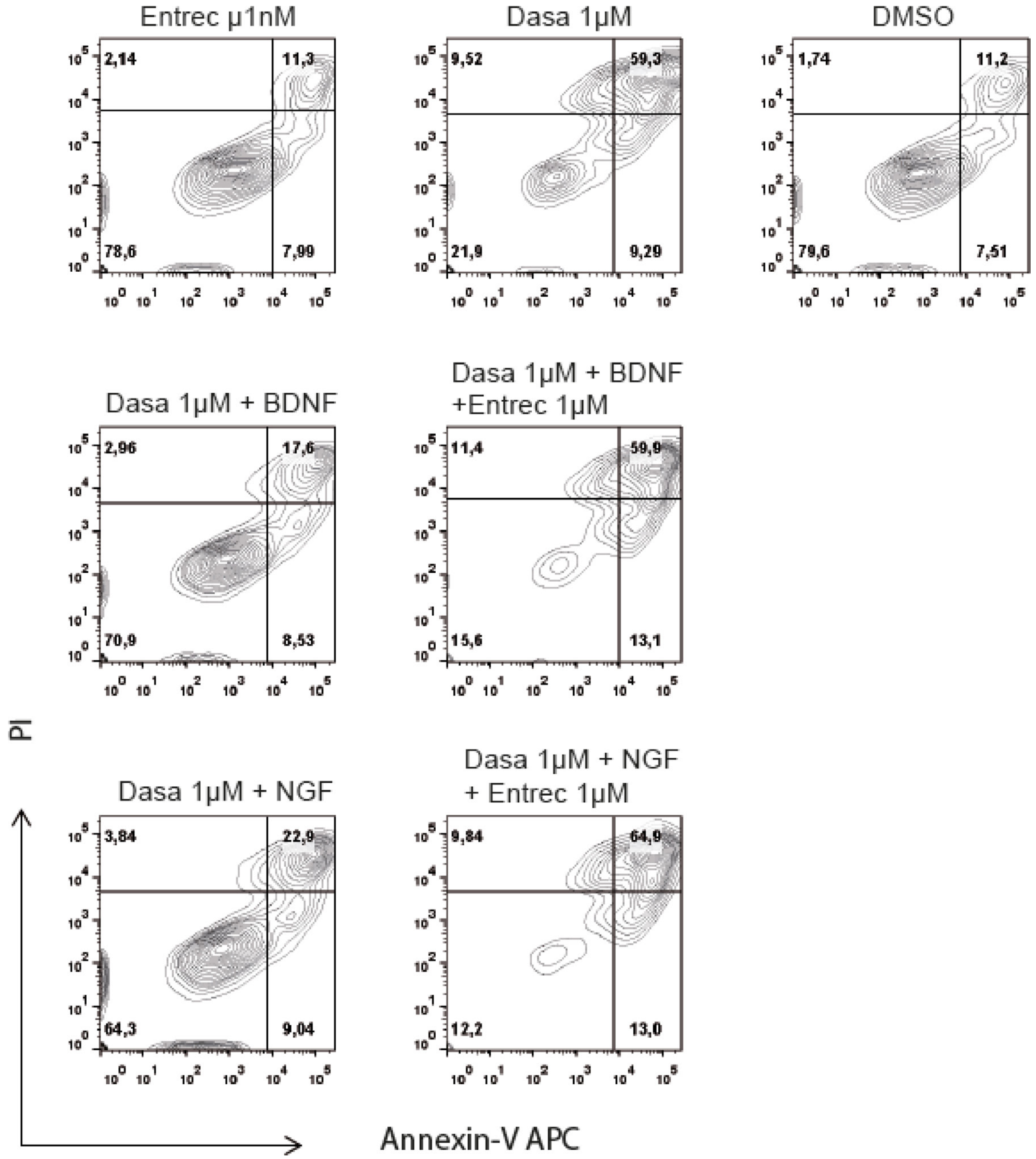

Supplementary Figure 5: Activation of TRKB was potent to rescue HMC-1 cells from cell death induced by KIT inhibition. Flow cytometry diagrams demonstrating apoptosis of HMC-1 cells with ectopic expression of TRKB after 48h treatment of inhibitors and BDNF (brain-derived neurotrophic factor, 100 ng/ml) stimulation. Note that activation of TRKB by its ligand BDNF was as potent as TRKA activation by NGF (100 ng/ml) to rescue HMC-1 cells from cell death induced by KIT inhibition. These rescue effect was efficiently blocked by entrectinib. Cell viability was analyzed using the Annexin-V assay. Dasa = dasatinib, Entrec = entrectinib, DMSO = dimethyl sulfoxide.

Supplementary Table 1: Quantitative RT-PCR analysis of gene expression in treated HMC-1 cells

Treatment groups	Expression of selected genes (fold)			
	EGR3	GDF15	KLF2	EGR1
DMSO	21.1	4.4	1.8	7.3
Dasa	1.0	1.0	1.0	1.0
Dasa + NGF	1269.4	3.9	2.2	10.3
Dasa+ NGF+ Entrec	1.4	1.8	1.0	1.1

Expression of selected genes in different groups was compared with that in the Dasa-group (fold). Dasa = dasatinib, NGF = nerve growth factor, Entrec = entrectinib.
